# Thermal and Herbicide Tolerances of Chromerid Algae and Their Ability to Form a Symbiosis With Corals

**DOI:** 10.3389/fmicb.2019.00173

**Published:** 2019-02-12

**Authors:** Leela J. Chakravarti, Andrew P. Negri, Madeleine J. H. van Oppen

**Affiliations:** ^1^Australian Institute of Marine Science, Townsville MC, QLD, Australia; ^2^AIMS@JCU, Australian Institute of Marine Science, College of Marine and Environmental Sciences, James Cook University, Townsville, QLD, Australia; ^3^College of Marine and Environmental Sciences, James Cook University, Townsville, QLD, Australia; ^4^School of BioSciences University of Melbourne, Parkville, VIC, Australia

**Keywords:** climate change, *Acropora*, ocean warming, diuron, symbiodiniaceae, *Chromera velia*, *Vitrella brassicaformis*, *Cladocopium goreaui*

## Abstract

Reef-building corals form an obligate symbiosis with photosynthetic microalgae in the family Symbiodiniaceae that meet most of their energy requirements. This symbiosis is under threat from the unprecedented rate of ocean warming as well as the simultaneous pressure of local stressors such as poor water quality. Only 1°C above mean summer sea surface temperatures (SSTs) on the Great Barrier Reef (GBR) can trigger the loss of Symbiodiniaceae from the host, and very low concentrations of the most common herbicide, diuron, can disrupt the photosynthetic activity of microalgae. In an era of rapid environmental change, investigation into the assisted evolution of the coral holobiont is underway in an effort to enhance the resilience of corals. Apicomplexan-like microalgae were discovered in 2008 and the Phylum Chromerida (chromerids) was created. Chromerids have been isolated from corals and contain a functional photosynthetic plastid. Their discovery therefore opens a new avenue of research into the use of alternative/additional photosymbionts of corals. However, only two studies to-date have investigated the symbiotic nature of *Chromera velia* with corals and thus little is known about the coral-chromerid relationship. Furthermore, the response of chromerids to environmental stressors has not been examined. Here we tested the performance of four chromerid strains and the common dinoflagellate symbiont *Cladocopium goreaui* (formerly *Symbiodinium goreaui*, ITS2 type C1) in response to elevated temperature, diuron and their combined exposure. Three of the four chromerid strains exhibited high thermal tolerances and two strains showed exceptional herbicide tolerances, greater than observed for any photosynthetic microalgae, including *C. goreaui*. We also investigated the onset of symbiosis between the chromerids and larvae of two common GBR coral species under ambient and stress conditions. Levels of colonization of coral larvae with the chromerid strains were low compared to colonization with *C. goreaui*. We did not observe any overall negative or positive larval fitness effects of the inoculation with chromerid algae vs. *C. goreaui*. However, we cannot exclude the possibility that chromerid algae may have more important roles in later coral life stages and recommend this be the focus of future studies.

## Introduction

Tropical reef-building corals provide a structural basis for one of the most productive and biodiverse ecosystem on Earth (Connell, 1978) that generates essential ecological goods and services (Moberg and Folke, 1999) in an otherwise oligotrophic environment. These foundation species form an obligate symbiosis with photosynthetic microalgae belonging to the family Symbiodiniaceae (LaJeunesse et al., 2018), which they rely upon for most of their energy requirements via the translocation of photosynthetic products from symbiont to host (Muscatine and Porter, 1977; Falkowski et al., 1984; Muscatine, 1990). Coral reefs are under serious threat from a range of anthropogenic pressures including ocean warming driven by the unprecedented release of anthropogenic carbon dioxide into the atmosphere (Hoegh-Guldberg, 1999; Pandolfi et al., 2011; Hughes et al., 2017), as well as local pressures such as a degradation of water quality through terrestrial run-off into coastal systems (Fabricius, 2005).

Reef-building corals live near their upper thermal limits (Berkelmans and Willis, 1999) and above this threshold the association between corals and their photosynthetic symbionts, Symbiodiniaceae, breaks down. Most evidence to-date points toward the excessive production of reactive oxygen species (ROS) from heat- and light-driven disruption to photosynthesis as the underlying cause of this breakdown (Lesser, 1997; McGinty et al., 2012), although more recent studies suggest changes in the nutrient exchange between both partners may also play a role (Wooldridge, 2009a,b; Wiedenmann et al., 2013; Pogoreutz et al., 2017). The dissociation between the Symbiodiniaceae and coral and resulting paling of coral tissues is a process known as coral bleaching (Glynn, 1984). Without their algae the coral host will eventually die, and thus coral bleaching is a phenomenon often leading to mass mortality (Glynn, 1984; Berkelmans et al., 2004). On the Great Barrier Reef (GBR), four mass bleaching events were triggered by unusually high summer ocean temperatures in 1998, 2002, 2016, and 2017, with the proportion of reefs in the latter experiencing four times more bleaching than previous events (Hughes et al., 2017).

Herbicide additions into the marine environment through terrestrial weed control and antifouling paints also pose a growing threat to marine life (Lewis et al., 2009). On the GBR, summer coincides with monsoonal rainfall (Lough, 2007; Kroon et al., 2012; Lough et al., 2015) and thus many reefs are prone to the simultaneous effects of herbicide and thermal stress. Increases in the intensity and frequency of rain and storm events associated with climate change lead to episodes of heightened exposure of coastal systems to terrestrial run-off (Noyes et al., 2009). Diuron is one of the most commonly applied herbicides in the catchments of the GBR (Shaw et al., 2010) and is of particular concern due to its long persistence, high mobility and potency (Owen et al., 2003; van Dam et al., 2012; Mercurio et al., 2016). Indeed, diuron is detected year-round in some parts of the GBR (Kennedy et al., 2012). Diuron targets photosystem II in the chloroplast of photosynthetic organisms by binding to the D1 protein. It inhibits electron transport (Jones et al., 2003) and chronic exposure has been directly linked to coral bleaching (Jones and Kerswell, 2003; Jones et al., 2003; Cantin et al., 2007). Furthermore, diuron exacerbates the negative effect of other stressors, such as elevated temperature, on coral reef species (Negri et al., 2011; van Dam et al., 2012).

The rapid rate of environmental change represents a mounting challenge for the stability and function of the ancient symbiosis between the Symbiodiniaceae and coral host, particularly given that thermal thresholds for corals are already regularly exceeded (Hughes et al., 2017). Shuffling to Symbiodiniaceae communities dominated by more thermally tolerant strains can increase coral bleaching tolerance by 1-1.5°C (Berkelmans and van Oppen, 2006) but such shifts are generally temporally unstable (Jones et al., 2008). Furthermore, some corals show high levels of symbiont fidelity (Baker, 2003; Fabina et al., 2012) and therefore have limited scope for symbiont shuffling. *Ex hospite* directed selection for thermally resistant Symbiodiniaceae that can subsequently be introduced into aposymbiotic corals has therefore been explored as an intervention strategy to assist corals in their adaptation to ocean warming (van Oppen et al., 2015; Chakravarti et al., 2017; Chakravarti and van Oppen, 2018).

In addition to the Symbiodiniaceae, there is a diversity of eukaryotes associated with corals that include the apicomplexan-related lineages (ARLs), many of which are largely uncharacterized (Clerissi et al., 2018), but can occur in high prevalence across coral groups (Kirk et al., 2013a,b; Kwong et al., 2018) Further, the discovery of apicomplexan-like algae with a functional photosynthetic plastid that live in close association with corals potentially opens new opportunities for assisted evolution to take advantage of alternative sources of phototrophic energy. Alternative photosymbionts include *Chromera velia, first* isolated from the coral *Plesiastrea versipora* in Sydney Harbor and formally described as an apicomplexan-like alga in the Phylum Chromerida (Moore et al., 2008). Another chromerid strain initially also identified as *C. velia* was isolated from the tropical coral *Leptastrea purpurea* at One Tree Island, southern GBR (Moore et al., 2008). This strain shares metabolic features and photosynthetic ability with *C. velia* and is phylogenetically closely related, but differs substantially in morphology, cell ultrastructure and life-history and was later formally described as *Vitrella brassicaformis* (Oborník et al., 2012). While most species in the Apicomplexa are parasitic protists and contain an unpigmented remnant chloroplast, the chromerids *C. velia* and *V. brassicaformis* contain a functional photosynthetic plastid, supporting the hypothesis that apicomplexans and dinoflagellates such as those belonging to the Symbiodiniaceae share a common ancestor (Gajadhar et al., 1991). Since their discovery, re-analyses of global microbial surveys found abundant apicomplexan-like eukaryotic sequences that were tightly associated with tropical corals, but in previous microbial surveys were mistaken as novel bacteria (Janouškovec et al., 2012). The association of chromerids and other apicomplexan-related lineages with healthy corals suggests a potentially mutualistic relationship between these algae and cnidarians, although further investigation into the frequency and extent of this association is needed.

Despite its apparent prevalence, no studies have investigated the environmental tolerance of the chromerids, and only two short-term laboratory studies have investigated the onset of symbiosis between chromerids and coral. In the first study, three new *C. velia* cultures, isolated from GBR corals were found to colonize two species of GBR coral, *Acropora digitifera* and *A. tenuis*; *C. velia* cells were harbored within the larval endoderm and ectoderm, up to 3 days after exposure of the larvae to the algae (Cumbo et al., 2013). These findings were interpreted as evidence for an endosymbiotic relationship between *C. velia* and corals. In contrast, other studies have indicated apicomplexan-related lineages are found on coral surfaces (Janouškovec et al., 2012) or exclusively in coral biogenous sediments and not in coral tissues (Mathur et al., 2018). The second study exposed larvae of the tropical coral, *A. digitifera*, to the *C. velia* strain that was isolated from *Plesiastrea versipora* in Sydney Harbor (Moore et al., 2008), and compared the transcriptomic response of the inoculated larvae with that of non-inoculated control larvae for up to 2 days after their introduction (Mohamed et al., 2018). Genes involved in a typical host response to harmful parasites were upregulated when corals were inoculated with *C. velia* that led the authors to conclude a non-mutualistic relationship exists between coral and *C. velia*. However, the uptake of *C. velia* by the coral host was not quantitatively measured, and the environmental differences at the collection locations of the *C. velia* and the coral could have contributed to the results of this study. Finally, the existence of host-symbiont specificity is well-documented for the coral-Symbiodiniaceae symbioses, and similar specificity may exist for strains of the chromerids (Rodriguez-Lanetty et al., 2004; LaJeunesse et al., 2010; Smith et al., 2017). The absence of data on the physiological response of the chromerids to adverse environmental conditions along with the limited number of experiments investigating the onset of symbiosis between chromerid algae and corals means that it remains uncertain whether the coral-chromerid association can function as a mutualism.

From an assisted evolution view-point, the ability of *C. velia* (and *V. brassicaformis*) to photosynthesise, the prevalence and association of apciocomplexan-like sequences with corals and the successful short-term uptake of *C. velia* by two coral species, warrants further investigation into the apicomplexans as coral symbionts. Here we investigate the tolerance of three cultured strains of GBR *C. velia* and one strain of *V. brassicaformis*, to temperature and diuron and both stressors simultaneously, and compare this to the tolerance of a culture of the globally distributed *Cladocopium goreaui* belonging to the family Symbiodiniaceae (formerly known as *Symbiodinium goreaui*, with an ITS2 designation of type C1) (Trench and Blank, 1987; LaJeunesse, 2005). This alga is a host-generalist, commonly found in association with corals on the GBR (LaJeunesse et al., 2003, 2004). We also investigate the stability of the association of each microalgal strain with larvae of two GBR coral species, as well as larval mortality, under heat, and herbicide stress to assess whether any differences in heat and herbicide tolerances in culture transfer to the coral host upon their inoculation. Our investigation explored the use of alternate symbionts, the Chromerida, as a way to enhance the environmental stress tolerance of corals in a rapidly changing ocean.

## Methods and Materials

### Experimental Microalgal Strains

*Cladocopium goreaui*, strain SCF055-01.10, was isolated from the coral *A. tenuis*, Nelly Bay, Magnetic Island, Australia (Table 1) (19°1006^′′^S, 146°50060^′′^E) in 2010 and a culture was maintained at 27°C and 65 ± 10 μmol photons.m^−2^.s^−1^ (Sylvania FHO24W/T5/865 fluorescent tubes) under a 14:10 light:dark cycle.

**Table 1:** Species, host species and site of origin of each microalgal strain used in this study.

**Strain identification**	**Species**	**Host species**	**Geographic origin**	**Original study**
SCF055-01.10	*Cladocopium goreaui* (formerly *Symbiodinium* goreaui: ITS2 type C1)	*Acropora tenuis*	Magnetic island (Central GBR)	Chakravarti et al., 2017
Mdig2 (named “*C. velia1”* in this study)	*Chromera velia*	*Montipora digitata*	Magnetic island (Central GBR)	Cumbo et al., 2013
Mdig3 (named “*C. velia2”* in this study)	*Chromera velia*	*Montipora digitata*	Magnetic island (Central GBR)	Cumbo et al., 2013
CvLp_vc08/1, CCMP3155 or CMS22 (named *Vitrella brassicaformis* in this study)	*Vitrella brassicaformis* (formally known as *C. velia)*	*Leptastrea purpurea*	One tree island (Southern GBR)	Moore et al., 2008; Oborník et al., 2012 and more
SCF055-02 (named “unknown chromerid” in this study)	Unknown (tentatively *V. brassicaformis*)	*Acropora tenuis*	Magnetic island (Central GBR)	This study

Note that this strain is the same used by Chakravarti et al. (2017), and was derived as a monoclonal culture from the heterogeneous culture, investigated in two prior studies (Howells et al., 2012; Levin et al., 2016), however, this strain was then referred to as *Symbiodinium goreaui* type C1 (Table 1).

An unidentified alga (strain SCF055-02) was obtained from the original heterogeneous *C. goreaui* culture that grew unexpectedly in a replicate culture from the coral extract (Table 1). This occurred during a mutagenesis experiment where 256 replicate cultures were exposed to the chemical mutagen ethyl methane sulphonate (EMS), based upon methods from Chaturvedi and Fujita (2006) and subsequently exposed to 100 μg L^−1^ of diuron. This experiment was designed to select for mutation(s) that conferred diuron tolerance in *C. goreaui*. The replicate cultures were checked monthly for any indication of growth, using light microscopy and pulse-amplitude modulated fluorometry. After approximately 1 year, one of 256 replicate culture vessels showed signs of live cells. After subsequent, continuous re-inoculation into freshly prepared, sterile culture media, Daigo's IMK for Marine Microalgae (Nihon Pharmaceutical Co., Ltd) containing diuron, this replicate culture remained alive and showed growth and was given the identification SCF055-02. This replicate was subsequently cultured with 30 μL^−1^ of diuron for 2.5 years, with monthly sub-culturing into fresh media. To investigate its genetic identity, we extracted DNA using the Wayne's method (Wilson et al., 2002) and amplified the 18S rDNA small subunit region using the universal eukaryotic Forward (ss5 – 5′- GGTTGATCCTGCCAGTAGTCATATGCCTTG - 3′) and Reverse (ss3 – 5′- GATCCTTCCGCAGGTTCACCTACGGAAACC - 3′) primers (Rowan and Powers, 1992; Cumbo et al., 2013). PCR amplification was performed using 2 ng of genomic DNA as the template and with 12.5 μL of mastermix (Qiagen) and 5 μL of each primer. Conditions for amplification were the same as in Cumbo et al. (2013). PCR products were Sanger sequenced in both directions at the Australian Genomics Research Facility (AGRF). Forward and reverse sequences were assembled in Sequencher (Version 5.4.5) and BLASTn (database: nucleotide collection nr/nt) searches of the assembled sequences were conducted at blast.ncbi.nlm.nih.gov.

The top 10 blast matches were all to the chromerids *C. velia* and *V. brassicaformis* (Table S1). Two GBR cultures of chromerids, Mdig2, and Mdig3, assumed to be *Chromera velia* (from Cumbo et al., 2013) were obtained (Table 1), and 18S sequences downloaded from Genbank (Accession numbers JN986789.1 and JN986790.1, respectively). One strain of *V. brassicaformis* was sourced [originally from Moore et al. (2008) and later described by Oborník et al. (2012)] and also 18S sequenced, as before. To identify our unknown chromerid as either *C. velia* or *V. brassicaformis* we aligned the four 18S chromerid sequences in Sequencher (version 5.2.4) and found only one base pair different between the unknown chromerid and *V. brassicaformis* and five and four base pairs different with Mdig2 and Mdig3, respectively (renamed *C. velia1* and *C. velia2* henceforth). Phylogenetic analyses were carried out in Mega (version 7.0.18) and were inconclusive due to the similarity of each of the four chromerid the sequences (Figure S1).

Microscopic observations revealed the most similar morphologies of SCF055-02 with *V. brassicaformis* compared to the two *C. velia* strains (Figure S2). Further genetic and morphological analyses are required to confirm this. For the purpose of this study we name SCF055-02 as the “unknown chromerid.” Thus, from here forth the four chromerid strains are described as *C. velia1* and *C. velia2* (strains Mdig2 and Mdig3, respectively), *V. brassicaformis* and the unknown chromerid strain (Table 1).

### *In vitro* Temperature and Diuron Sensitivity of *Cladocopium goreaui* and the Chromerids

Firstly, to test the sensitivity of *C. goreaui* and chromerid strains to temperature, diuron and their combined effects, we carried out dose-response toxicity assays using 11 diuron concentrations (0, 0.3, 1, 3, 10, 30, 100, 300, 1,000, 3,000 and 5,000 μg L^−1^) and four temperatures (27°C control, 30°C, 31°C and 32°C, Table S2). This temperature range was chosen based on previous findings that the strain of *C. goreaui* used in this study cannot survive at 31°C or beyond for a prolonged period of time (Chakravarti et al., 2017). The highest temperature treatment of 32°C was chosen because the thermal sensitivities of the chromerids were unknown (but see Visser et al., 2012). Experiments took place using four temperature-controlled environmental chambers (Steridium, er-rh-500) with 65 ± 10 μmol photons.m^−2^.s^−1^ (Sylvania FHO24W/T5/865 fluorescent tubes) under a 14:10 light:dark cycle and temperature measurements were recorded every 10 min, using a data logger (HOBO Pendant, Table S2).

Cells were pre-acclimated to the four temperatures, for either 10 or 20 days prior to dosing with diuron, to test whether the duration of temperature pre-exposure had an effect on thermal sensitivity, or an interactive effect with diuron. Ten and 20-day pre-acclimation durations were based on previous experiments with the *C. goreaui* strain used in this study that demonstrated no negative photosynthetic effects after 13 days at 32°C (Levin et al., 2016) but negative physiological effects of 31°C after 17 days (Chakravarti et al., 2017).

A stock solution of diuron was prepared by dissolving analytical grade diuron in 100% ethanol. The 11 concentrations were prepared at double concentration in IMK medium, using the stock diuron solution as well as an ethanol solvent control solution (carrier only, 0.003% (v/v) final concentration), representing the highest ethanol concentration in the 5,000 μg L^−1^ diuron treatment. Fifty μL of each of the 11 diuron concentrations and solvent control were transferred into triplicate black, clear-bottom 96-well culture plates (Costar, Corning^®^, Sigma-Aldrich) in a randomized well-design. Temperature pre-acclimated cultures were pelleted (295 g/2,000 rpm, 5 min), media removed, and cells resuspended in fresh IMK media at a cell density twice that required for the diuron dosing (3,200,000 cells mL^−1^). Fifty μL of each culture were subsequently added to each well to give a final concentration of 1,600,000 cells mL^−1^ and with a final volume of 100 μL. This resulted in nine replicate wells for each microalgal culture, spread across triplicate plates. Plates were placed in their respective temperature conditions under 60 μmol photons m^−2^s^−1^ for 48 h before chlorophyll a fluorescence measurements were taken. The effective quantum yield in an illuminated plant [ΔF/F${}_{\text{m}}^{\prime }$ = (F${}_{\text{m}}^{\prime }$ – F′)/ F${}_{\text{m}}^{\prime }$] provides an estimate of the efficiency of photochemical energy conversion within photosystem II under a given light intensity (Genty et al., 1989). The reversible binding of photosystem II herbicides to the D1 protein in photosystem II results in an acute and temporary reduction in ΔF/F_m_' (Jones and Kerswell, 2003). The maximum quantum yield [F_v_/F_m_ = (F_m_ – F_0_) / F_m_] is equivalent to the proportion of light used for photosynthesis by chlorophyll when all reaction centers are open (Genty et al., 1989) and reductions in Fv/Fm indicate inactivation and/or photo-oxidative damage to photosystem II (chronic photoinhibition) (Schreiber, 2004). Finally, we measured the maximum excitation pressure over photosystem II, Q_m_ = 1− [(ΔF/F_m_′)/ F_v_/F_m_)], where values close to zero indicate that even during periods of the maximum irradiance most reaction centers remain open, suggesting that photosynthetic rates are light-limited. However, values close to 1.0 indicate that under maximum irradiance most of the photosystem reaction centers are closed, suggesting photoinhibition (Iglesias-Prieto et al., 2004).

Maximum quantum yield measurements were taken 1 h before the end of the dark cycle, and effective quantum yield measurements were attained by subsequently pre-acclimating plates to 6 min of an actinic light of two (PAR = 20 units) before applying a saturation pulse, methods based on (Schreiber et al., 2007). Measurements for effective quantum yields were carried out twice and a mean value for each replicate obtained (nb. a preliminary study showed that there were no significant differences between mean effective quantum yield values of cells exposed to 6 min of actinic light once and cells exposed to 6 min of actinic light six times, with saturation pulses in between).

The results from 0 μg L^−1^ of diuron in these experiments were used to investigate the effects of temperature alone on the photochemistry of these microalgae. Therefore, results here reflect a total of 12 and 22 days temperature exposure. The maximum quantum yields (F_v_/F_m_) and pressure over photosystem II, (Q_m_) are reported only for the effect of temperature on the different microalgal strains.

### Coral Larval Inoculation With *Cladocopium goreaui* and Chromerids

To determine the relative colonization abilities of *C. goreaui* and the four chromerid strains, as well as their effect on the coral host, we introduced the microalgae to aposymbiotic larvae of two coral species, *Acropora tenuis* and *Acropora millepora*. Colonies of *A. tenuis* were collected from Falcon Island (18°46' E 146°32' S) in November 2017, while colonies of *Acropora millepora* were collected from Backnumbers Reef (18°29'264”E 147°09'174”S) in December 2017. Both species were kept in the National Sea Simulator at the Australian Institute of Marine Science (Townsville, Australia) for 3 days before the full moon. Following spawning, gametes were collected from six colonies of each species and gametes from the same species were mixed, using approximately equal sperm concentrations (~10^6^ mL^−1^) for fertilization. Resulting larvae were kept in aerated, flow-through 0.4 μm filtered seawater, for 12 and 37 days before the experiment for *A. tenuis* and *A. millepora*, respectively.

*Cladocopium goreaui* and the chromerid cultures were pre-acclimated to three temperature treatments of 27°C, 30°C and 31°C (Table S2) for 2 weeks before being introduced to the coral larvae. Larvae were pre-acclimated for 3 days to each temperature treatment (Table S3). From previous observations, the uptake of *C. goreaui* at 31°C for *A. tenuis* and *A. millepora* has been minimal and thus we chose 31°C as the maximum temperature treatment for this *in hospite* experiment. Ten larvae were added to each well of triplicate, six-well plates (Corning, Sigma-Aldrich) containing 9 mL of *C. goreaui* or chromerid algae in filtered seawater, at a concentration of 15,000 cells mL^−1^ and containing either 30 μg L^−1^ of diuron or 0 μg L^−1^ diuron. Plates were placed at the three temperatures under 60 μmol photons m^−2^s^−1^. This experimental setup resulted in 180 larvae *per* diuron treatment *per* temperature, for each coral species. Two days later, a near-full water change was conducted in each well. Subsequently, every 2 days, the salinity in one randomly chosen well of each plate was measured and water changes were conducted for all wells if the salinity had increased.

Fourteen days after their exposure to *C. goreaui* and the chromerids, one larva *per* well was removed, placed on a microscope slide and viewed under a fluorescent microscope (Zeiss Axioscop2 molt plus) using the filter (Zeiss F. set09 (FITC), ex 495 nm em 517 nm) allowing the algal cells to be clearly differentiated from the host cells by observing the red autofluorescence of chlorophyll within the algae. For each temperature, diuron treatment and larval species, the total number of algal cells hosted by the larvae was counted (*n* = 18 larvae across three triplicate plates), with larvae containing one or more algal cells being recorded as “colonized.” The proportion of colonized larvae was also calculated (*n* = 3: the proportion of colonized larvae that were removed from each plate, across triplicate plates). Finally, the number of larvae remaining in each well at each time point was recorded to assess mortality (*n* = 18 wells across triplicate plates). Any larvae that had metamorphosed were removed from the wells, but still counted as alive. After 14 days, remaining larvae were fixed in 2.5% glutaraldehyde and stored at 4°C for visualization on a confocal microscope.

### Confocal Microscopy

Slides were prepared with larvae, using a mounting solution of 90% glycerol, 10% phosphate buffer solution (PBS) and were visualized using a laser scanning confocal microscope (Zeiss 710) using an EC Plan-Neoflaur 20x/0.50 M27 objective with a pinhole (depth resolution) of 28 μm, emission wavelengths of 435–726 nm with the following excitation wavelengths and intensity: 405 nm: 60%, 561 nm: 45% and 488 nm: 55%. The master gain was set at 476.

### Statistical Analyses

#### In vitro

To test the effects of diuron concentration and the interaction of diuron and temperature on the photochemistry of *C. goreaui* and the chromerids, effective quantum yield data were fitted with a variable slope log(dose) vs. response curve and the EC50 values (the concentration of diuron that gives half-maximal response) were interpolated from the non-linear fits. Confidence intervals (95%) were calculated for each mean EC50 value and compared for each microalgal strain. We considered the overlap of 95% confidence intervals between means as the means not being significantly different and *vice versa*. Analyses were carried out using GraphPad Prism (v. 7.03). The effects of temperature and strain on photophysiological traits were tested using linear mixed effects models with plate as a random factor. Analyses were performed in R (v. 3.4.1; R Core Team, 2017) using the packages, nlme (v. 3.1-131) and multcomp (v. 1.4-8).

#### In hospite

To test the effects of microalgal strain, diuron and their interaction on the uptake of microalgae and mortality in two species of coral larvae, we carried out general linearised models with Poisson (uptake) and binomial (mortality) distributions. Where there were no significant interactions between strain and diuron, the interaction term was removed and run as an additive model; model choice was confirmed using the Akaike Information Criterion (AIC). *Post-hoc* analyses were carried out using Tukey's tests (*p* > 0.05) and when a significant strain by diuron interaction was evident, a planned comparison matrix was carried out. This involved comparing uptake/mortality between all strains on larvae inoculated with each strain. For mortality data, mean proportion values were calculated from differing replicate numbers, therefore replicate weights were specified in the model. Separate analyses were carried out for both traits (larval uptake and larval mortality) at the three temperature conditions, 27, 30, and 31°C. Analyses were carried out in R (v. 3.4.1; R Core Team, 2017) using the following packages; lme4 (v. 1.1-17), car (v. 3.0-0), MASS (7.3-47), multcomp (v. 1.4-8), and MuMIn (v. 1.42.1). Raw data are supplied in Supplementary Material (Tables S7–S11).

## Results

### *In vitro* Experiments

A series of *in vitro* experiments demonstrated that temperature alone, diuron, alone and the simultaneous exposure of temperature and diuron affected the photochemistry of all microalgae tested and that there were striking differences in response between strains.

#### Effect of Temperature on Photochemistry

**Figure 1:**
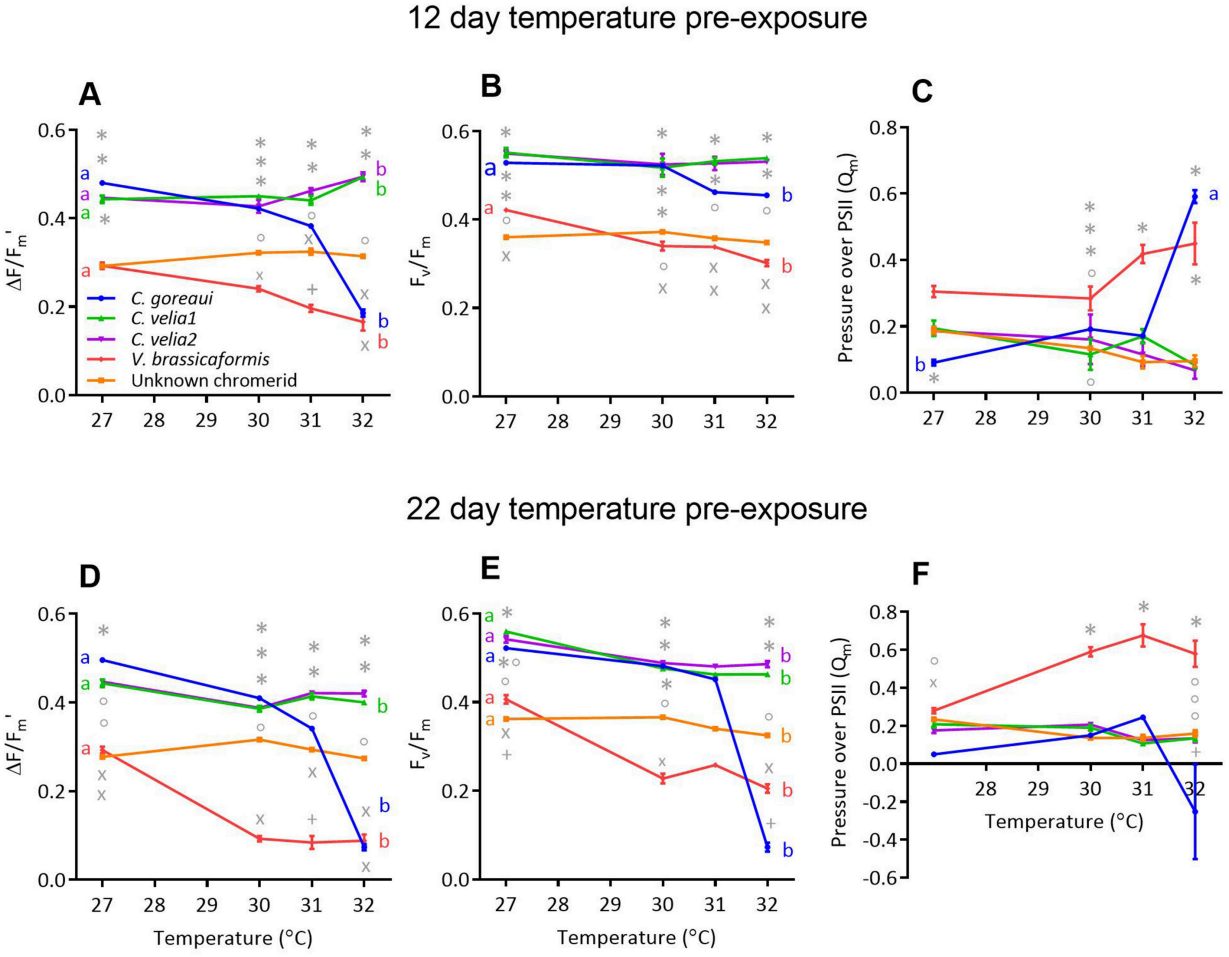
The effect of 12 days **(A–C)** and 22 days **(D–F)** of exposure to different temperatures on the **(A,D)** effective (ΔF/F_m_') and **(B,E)** maximum (F_v_/F_m_) quantum yields and **(C,F)** pressure over photosystem II of *Cladocopium goreaui* and four chromerid strains. Different symbols represent statistically significant differences in photochemistry between strains within the same temperature treatment. Different lowercase letters represent statistically significant differences between photophysiological traits at 27 and 32°C Data points represent means (*n* = 9) ± SE.

Strain, temperature and their interaction had a significant effect on microalgal effective (ΔF/F_m_′) and maximum (F_v_/F_m_) PSII quantum yields and pressure over photosystem II (Q_m_) after both 12 and 22 days of temperature exposure (Table S4). *Post-hoc* comparisons indicated that there were differences in both ΔF/F_m_′ and F_v_/F_m_ at the control temperature of 27°C between the microalgal strains (Figure 1); *Cladocopium goreaui, C. velia1, C. velia2* all had significantly greater mean yields compared to *V. brassicaformis* and the unknown chromerid, that were up to 60% greater in ΔF/F_m_′ and 65% in F_v_/F_m_ (max *p* < 0.01). After both durations of temperature exposure, *C. goreaui* experienced the largest decline in both F_v_/F_m_ and ΔF/F_m_ ′ at the highest temperature treatment, falling by as much as 85–86% after 22 days and 14–57% after 12 days at 32°C, respectively (Figure 1B, max *p* < 0.01). This was accompanied by an 85% increase in mean Q_m_ after 12 days temperature exposure at 32°C (Figure 1C, *p* < 0.01), results that were no longer apparent after 22 days due to ΔF/F_m_′ and F_v_/F_m_ becoming equally low (Figure 1F, *p* = 0.06).

ΔF/Fm′ and Fv/Fm also decreased for *V. brassicaformis* with increasing temperature (Figures 1A,B,D,E, *p* < 0.01) and this species exhibited the highest Q_m_ values at 31°C after 12 days (Figure 1C, max. *p* = 0.046) and the highest at 30, 31, and 32°C after 22 days [Figure 1F, max. *p* < 0.01)]. *C. velia1, C. velia2* and the unknown chromerid on the other hand were able to maintain stable ΔF/Fm′ and Fv/Fm and Q_m_ value across the temperature conditions after 12 days of exposure (Figures 1A–C). After 22 days of exposure at 32°C, there was a small but statistically significant decrease by 9.6% in ΔF/F_m_′ for *C. velia1* compared to 27°C (Figure 1D, *p* < 0.01) while all three strains exhibited statistically significant decreases, by 17%, at the most, in F_v_/F_m_ at 32°C compared to 27°C (max. *p* < 0.01).

#### Effect of Diuron on Photochemistry

**Figure 2:**
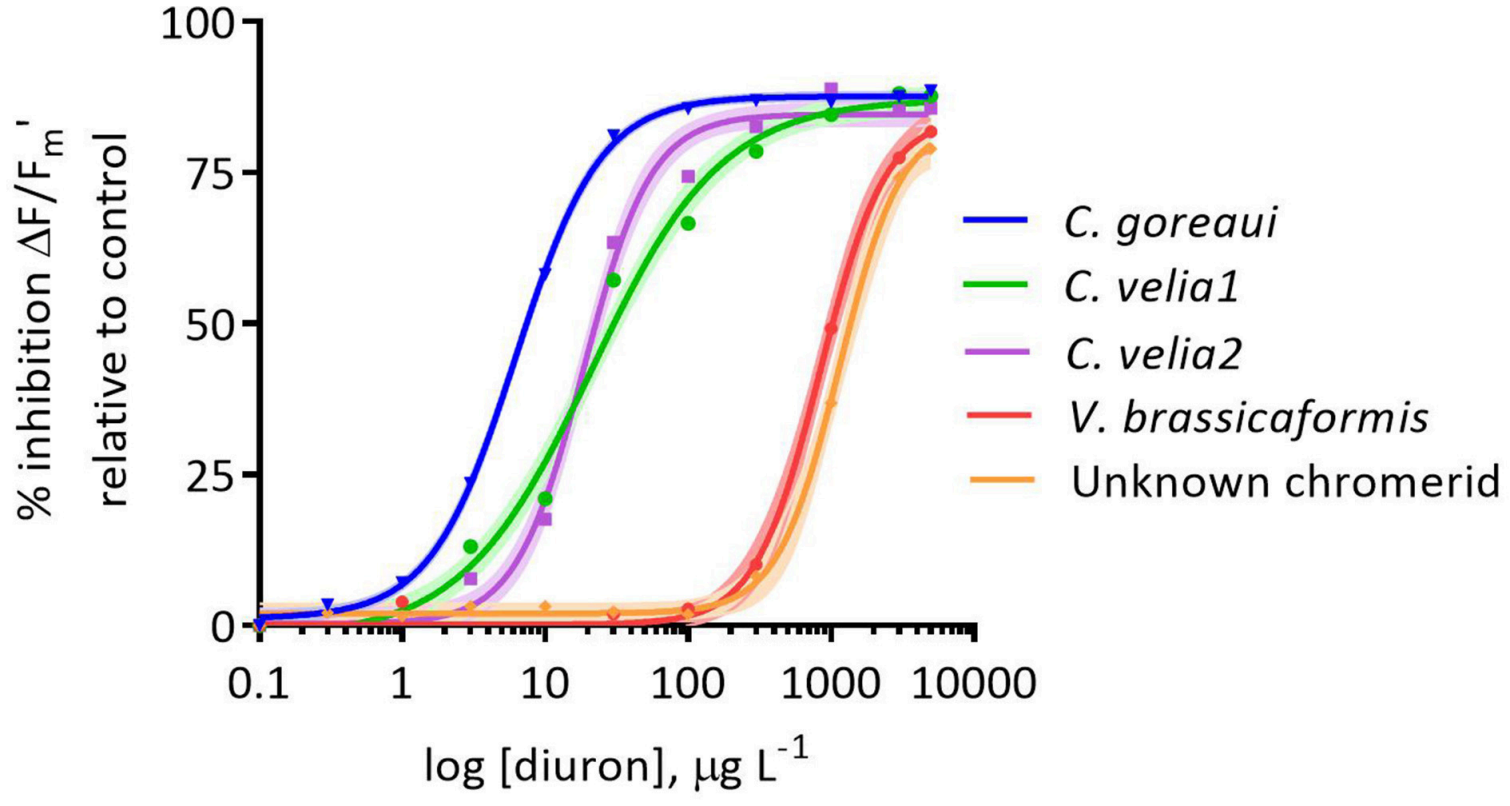
Dose-response of the percentage inhibition of the effective (ΔF/F_m_′) quantum yields of *Cladocopium goreaui* and four chromerid strains exposed for 48 h to diuron at 27°C. Inhibition is relative to the quantum yield values at 0 μg L^−1^of diuron. Curves are fitted with non-linear regressions. Data points represent means (*n* = 9). The narrow-shaded areas represent confidence interval (95%) bands.

There were significant differences in the effect of diuron on ΔF/F_m_′ between different microalgal strains (Table 2). *Cladocopium goreaui* was the most sensitive species, exhibiting the lowest EC50, approximately 4-fold and 3-fold lower than the EC50 values for *C. velia1* and *C. velia2* respectively (Figure 2, Table 2). *Vitrella brassicaformis* and the unknown chromerid were highly insensitive to diuron (Figure 2), requiring over a 130-fold higher diuron concentration for 50% ΔF/Fm′ inhibition in comparison to *C. goreaui* (Table 2).

**Table 2:** EC50 values and 95% confidence intervals (CI) for effective ($\Delta F/F_{\text{m}}^{\prime }$) quantum yields after 48 h of exposure to diuron at 27°C.

	**ΔF/Fm'**	
**Microalgal strain**	**EC50 (μg L^**−1**^)**	**CI range**
*Cladocopium goreaui*	7.4	7.2–7.7
Unknown chromerid	1,365	1,226–1,551
*Chromera velia1*	30	27–35
*Chromera velia2*	22	21–25
*Vitrella brassicaformis*	1,024	930–1,134

#### Effect of Combined Diuron and Temperature on Photochemistry

**Figure 3:**
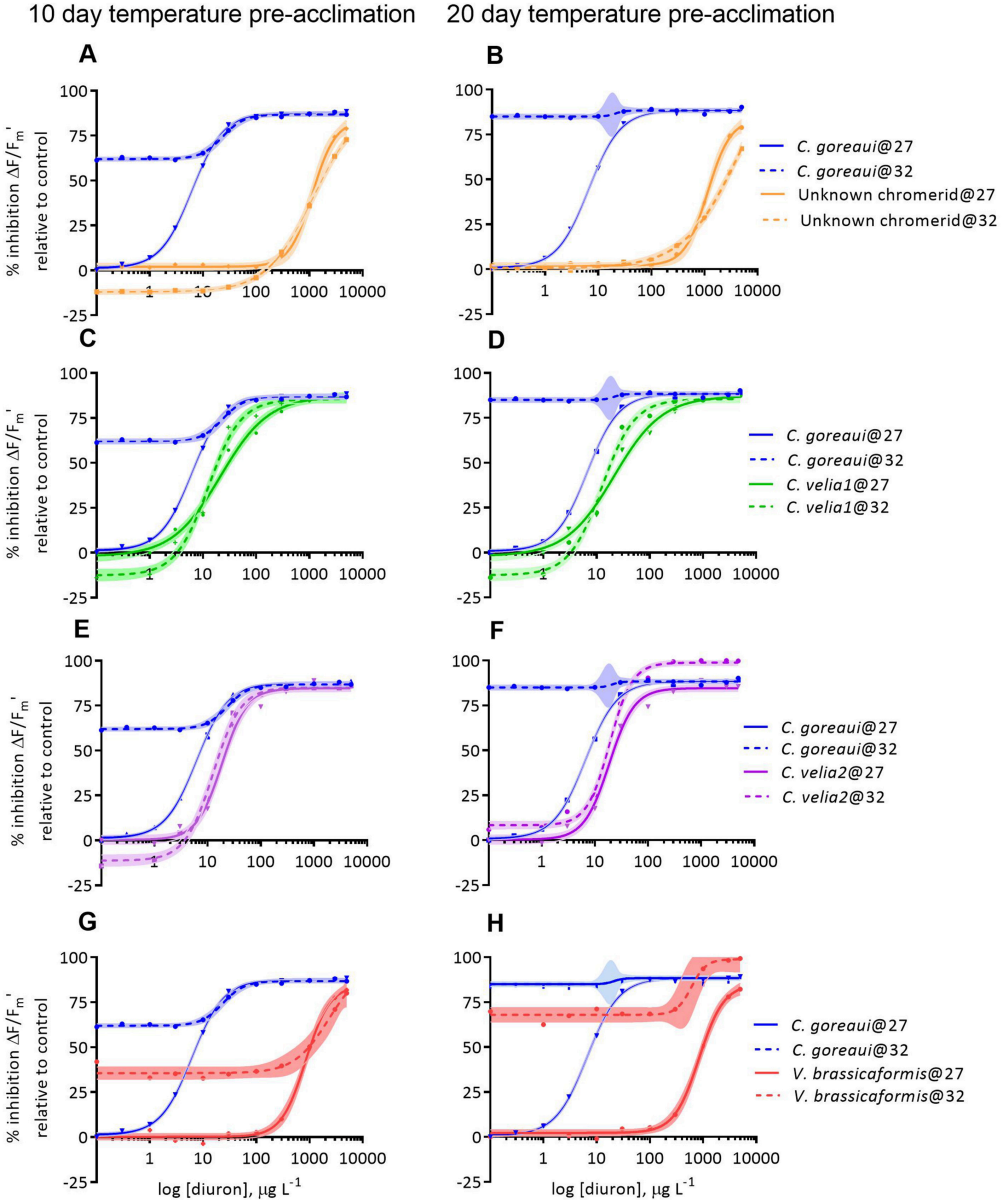
Dose-responses to diuron of the percentage inhibition of effective (ΔF/F${}_{\text{m}}^{\prime }$) quantum yields of *Cladocopium goreaui* and four strains of chromerids at 27 and 32°C, after 10 **(A,C,E,G)** and 20 days **(B,D,F,H)** of exposure to each temperature before being exposed to diuron for 48 h and thus spending a total of 12 and 22 days exposed to the different temperature conditions. Inhibition is relative to the quantum yield values at 0 ug L^−1^ of diuron and 27°C for each strain. Curves are fitted with non-linear regressions. Shaded areas represent confidence interval (95%) bands.

Temperature significantly affected the diuron sensitivity of ΔF/Fm′ across all of the microalgal strains after 12 days of exposure to 32°C (Figure 3, Table 3). *Cladocopium goreaui* experienced more than 50% inhibition in ΔF/F_m_ ′ at 32°C before any diuron additions (Figure 3A) and thus an EC50 value was not calculated (Table 3). The ΔF/F${}_{\text{m}}^{\prime }$ of *V. brassicaformis* was also strongly inhibited by temperature in the absence of diuron and this inhibition was greater after 22 d of elevated temperature in comparison to the 10 d exposure (Figures 3G,H, Table 3). The negative effects of diuron on ΔF/F${}_{\text{m}}^{\prime }$ increased at 32°C in *C. velia1* and *C. velia2* with EC50s reached at 18–43% lower diuron concentrations, and these negative impacts were also greater for the longer 22 d exposures (Figures 3C–F, Table 3). Interestingly the unknown chromerid became significantly less sensitive to diuron at elevated temperature as shown by the increase in EC50s at 32°C by 25 and 61% after 10 and 22 days, respectively (Figures 3A,B, Table 3). At low diuron concentrations the unknown chromerid, *C. velia1* and *C. velia2* all exhibited higher photosynthetic efficiencies (were less inhibited) at 32°C than at 27°C (Figures 3A,C–E).

**Table 3:** EC50 values and 95% confidence intervals (CI) for effective (ΔF/F${}_{\text{m}}^{\prime }$) quantum yields after 10 and 20 days of pre-acclimation to 27 or 32°C and a further 48 h post diuron-dosing and % changes in EC50 values with temperature.

**Temp. pre-acclimation (days)**	**Microalgal strain**	**27**^****°****^**C**		**32**^****°****^**C**		**% change**
		**EC50 (μg L^**−1**^)**	**CI range**	**EC50 (μg L^**−1**^)**	**Upper CI**	
10	*Cladocopium goreaui*	7.4	7.2–7.7	already > 50% inhibited		NA
20		7.9	7.6–8.1	already > 50% inhibited		NA
10	Unknown chromerid	1,365	1,702–1,551	1,702	1,578–1,832	+25
20		1,365	1,226–1,551	2,592	2,292–2,892	+61
10	*Chromera velia1*	30	19–35	19	17–21	−37
20		30	27–35	17	17–21	−43
10	*Chromera velia2*	22	18–25	18	16–20	−18
20		23	21–25	17	16–19	−26
10	*Vitrella brassicaformis*	1,024	971–1,134	971	561–1,505	−5
20		990	899–1,097	already > 50% inhibited		NA

*Blue highlighted % change values correspond to an increase in EC50 values with elevated temperature, while while red highlighted % change values correspond to a decrease in EC50 values with elevated temperature*.

### *In hospite* Experiments

A series of *in hospite* experiments involving the introduction of the five microalgal strains to coral larvae of two species, *Acropora tenuis* and *Acropora millepora*, revealed differences in their uptake capability and mortality under diuron and with no diuron, across three temperature treatments.

#### Acropora tenuis

##### Larval uptake

At 27 and 30°C the mean larval uptake of microalgae by *Acropora tenuis* was significantly affected by microalgal strain, diuron and their interaction. Only strain affected larval uptake at 31°C, although the means were low and differences were minimal (Table S5).

**Figure 4:**
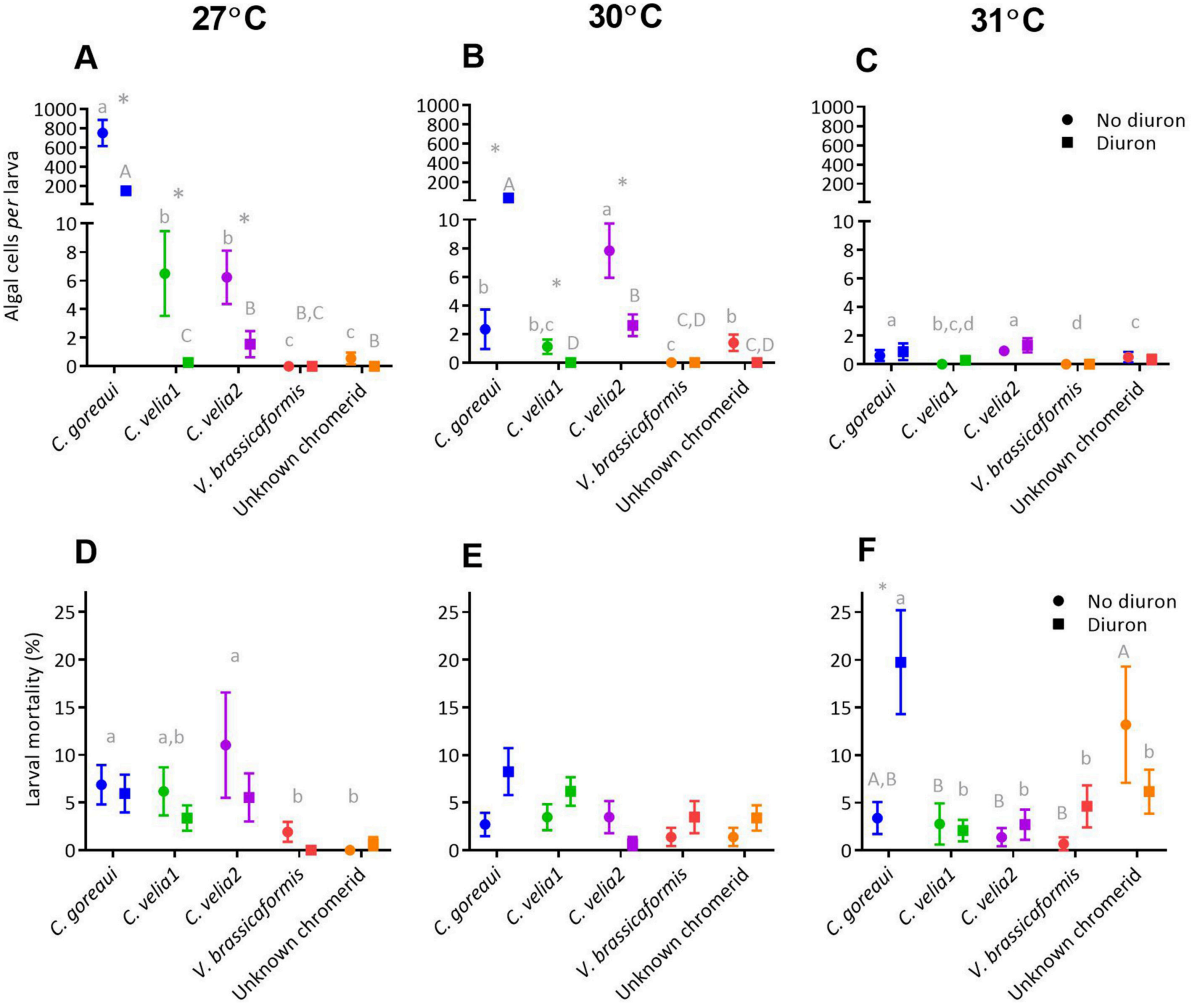
The effect of microalgal strain and diuron (30 μg L^−1^ diuron) on the uptake of microalgae and mortality of *Acropora tenuis* larvae at three temperature conditions, 27°C (**A** and **D**), 30°C (**B** and **E**) and 31°C (**C** and **F**) at day 14 of the experiment. Different lower-case letters represent statistically significant differences between strains in the absence of diuron, while upper-case letters represent statistically significant differences between strains under 30 μg L^−1^ of diuron. ^*^Symbol represents a significant effect of diuron within a strain (Tukeys tests, *p* < 0.05). Data points represent means ± standard error.

*Post-hoc* analyses revealed that at 27°C the uptake of *C. goreaui* by larvae was more than 115 times greater than the next most successful chromerids, *C. velia1* and *C. velia2* (Figure 4A, *p* < 0.01). Diuron significantly reduced the uptake of *C. goreaui, C. velia1* and Mdig 3 at 27°C (Figure 4B, *p* < 0.01), a trend that continued at 30°C for *C. velia1* and *C. velia2*. In contrast *C. goreaui* uptake was 15 times greater in the presence of diuron 30°C (Figure 3B, *p* < 0.01).

##### Larval mortality

At 27°C, both strain and diuron had statistically significant effects on larval mortality (Figure 4D, Table S5), whereby the three strains that had the highest uptake, *C. goreaui, C. velia1* and Mdig 3, also exhibited the greatest larval mortality, compared with the remaining strains (Figure 4D, *p* < 0.03). At 30°C, only the presence of diuron significantly affected larval mortality (Table S5), with an overall trend of increased mortality with diuron (Figure 4E), again reflecting a similar trend to larval uptake. At the highest temperature treatment of 31°C, larval mortality was significantly affected by algal strain, diuron and their interaction (Figure 4F, Table S5) and did not follow larval uptake trends. Specifically, under 0 μg/L of diuron, larvae exposed to the unknown chromerid had the highest mean mortality, that was 10% greater than the next highest, *C. goreaui* (Figure 4F). Under 30 μg L^−1^ of diuron, larvae with *C. goreaui* experienced 20% mortality, which was significantly higher than larvae exposed to and colonized with the remaining microalgal strains (Figure 4F, *p* < 0.01). Furthermore, larvae harboring *C. goreaui* were the only treatment at 31°C to be significantly affected by diuron (Table S5), evidenced by an increase in mortality by 8% in the presence of diuron (Figure 4F, *p* < 0.01).

#### Acropora millepora

##### Larval uptake

**Figure 5:**
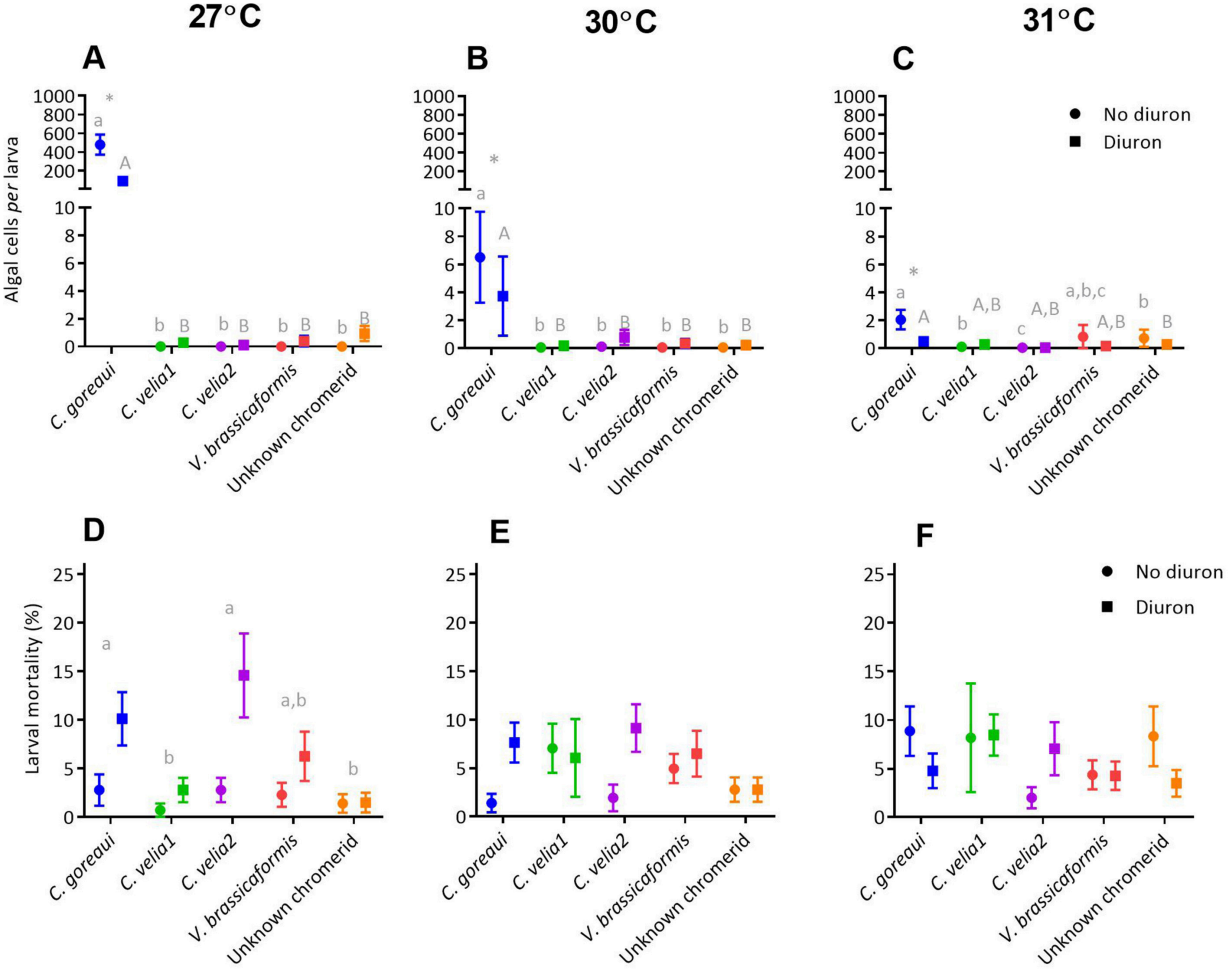
The effect of microalgal strain and diuron (30 μg L^−1^ diuron) on the uptake of microalgae and mortality of *Acropora millepora* larvae at three temperature conditions, 27°C (**A** and **D**), 30°C (**B** and **E**) and 31°C (**C** and **F**) after 14 days. Different lower-case letters represent statistically significant differences between strains in the absence of diuron, while upper case letters represent statistically significant differences between strains under 30 μg L^−1^ of diuron. ^*^Symbol represents a significant effect of diuron within a strain (Tukeys tests, *p* < 0.05). Data points represent means ± standard error.

At all temperature treatments, the mean uptake of microalgae by *Acropora millepora* larvae was significantly affected by microalgal strain, diuron and their interaction (Table S6). *Post-hoc* analyses revealed that such effects were most likely driven by *C. goreaui* where mean uptake of *C. goreaui* at 27 and 30°C was significantly greater than the mean uptake of the chromerids, under both diuron and no diuron conditions (Figures 5A,B, max. *p* < 0.01); chromerids all showed minimal colonization capacity, with larvae in these treatments harboring less than five symbionts *per* larva. Diuron significantly reduced the uptake of *C. goreaui* at all three temperature treatments (Figures 5A–C, max. *p* = 0.005), the greatest being a five times reduction in symbionts *per* larva at 27°C (Figure 5A, *p* < 0.01).

##### Larval mortality

At 27°C, both strain and diuron had statistically significant effects on larval mortality (Table S6), with a general trend of increased mortality under diuron conditions (Figure 5D). *Post-hoc* analyses indicated that larvae in the presence of *C. goreaui* and *C. velia2* had the highest mean mortalities (Figure 5D). At 30°C there was a significant effect of diuron and the interaction of strain and diuron on larval mortality (Table S6). Although *post-hoc* analyses were unable to identify specific differences in pairwise comparisons, trends show an overall increase in mortality with diuron, especially for *C. goreaui* and *C. velia2*, where mean mortality increased by over 7% (Figure 5E). At 31°C there was a significant effect on the interaction of strain and diuron on larval mortality (Table S6). Although *post-hoc* analyses were unable to disentangle these differences, trends indicate that mean mortalities surprisingly decreased with diuron for those larvae exposed to *C. goreaui*, and the unknown chromerid, while larvae with *V. brassicaformis* had increased mortality in the presence of diuron (Figure 5F).

## Discussion

**Figure 6:**
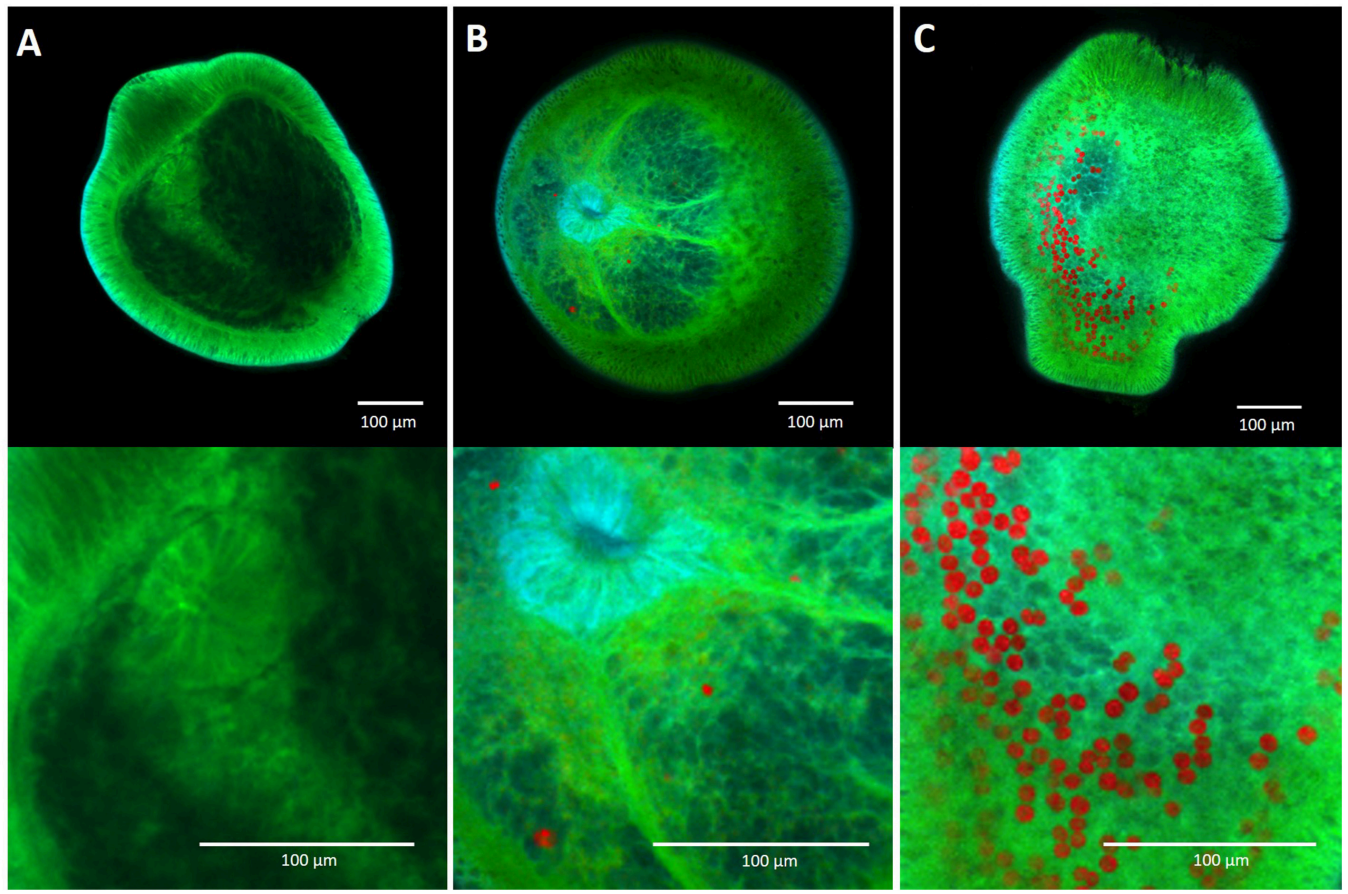
Confocal microscopy images of aposymbiotic *Acropora millepora* larvae **(A)**, larvae containing chromerid cells **(B)** and larvae containing *Cladocopium goreaui* cells **(C)**, 14 days post microalgal introduction to the larvae. Red pigmentation represents the chromerid and *C. goreaui* cells, while larval autofluorescence is represented in green.

The thermal and herbicide tolerances of the four chromerid strains was highly variable and differed substantially to that of the known photosymbiont of corals, *Cladocopium goreaui*. Three of the four chromerid strains exhibited high thermal tolerances and two strains had exceptional herbicide tolerances, greater than observed for other photosynthetic microalgae. Although coral larvae were able to take up each of the four chromerid strains, colonization was low in comparison to that of *C. goreaui* (Figure 6).

### The Effect of Temperature on Free-Living Microalgal Photochemistry

The thermal tolerance of photochemistry was highly variable between the microalgal strains. *Cladocopium goreaui* was the most sensitive, with negative effects of increasing temperature that were particularly severe with longer exposure. The impairment of photosynthetic function in the Symbiodiniaceae with elevated temperature is well-documented, often occurring above 30°C (e.g., Coles and Jokiel, 1977; Iglesias-Prieto et al., 1992; Hill et al., 2004; Takahashi et al., 2009) and has previously been shown in this same isolate (Chakravarti et al., 2017). The photosynthetic apparatus is susceptible to heat stress. In the Symbiodiniaceae moderate heat stress can result in photoinhibition of the chlorophyll *a* and *c* containing protein complex, photosystem II (PSII) (Warner et al., 1999), whereby heat (combined with light) disrupts the electron transport system (Jones et al., 1998) through several proposed mechanisms. These include the production of highly reactive singlet oxygen produced by PSII, that in turn causes further disruption to the electron transport system, photosynthetic pigments, proteins and thylakoid membranes (Hideg et al., 1994; Niyogi, 1999; Telfer et al., 1999; Krieger-Liszkay, 2004). Photoinhibition occurs when the rate of photodamage to PSII exceeds the rate of its repair (Warner et al., 1999; Takahashi et al., 2004). The dramatic drop in photosynthetic efficiencies and the increase in maximum pressure over PSII (Qm) observed for *C. goreaui* indicates severe photoinhibition for temperatures above 30°C in this study.

The two strains least affected by elevated temperature were the two *Chromera velia* strains, *C. velia1* and *C. velia2*, where only small decreases in photosynthetic efficiencies were observed after 22 days at 32°C. Photosynthesis in *C. velia* has been described as highly efficient despite representing a basic system, i.e., a simple composition of pigments that includes only chlorophyll (Chl) *a*, violaxanthin and a novel isofucoxanthin-like carotenoid (as major components) and a lack of any accessory pigments (Moore et al., 2008). Violaxanthin has been shown to act as a key factor in efficient photoprotection in *C. velia* through the unusually fast de-epoxidation of vioxlaxanthin (Kotabová et al., 2011). In agreement, increases in violaxanthin in *C. velia* (isolate from Moore et al., 2008) grown under high light conditions resulted in a doubling in non-photochemical quenching values, reflecting the dissipation of non-photochemical energy, and 30% reduction in excitation pressure over PSII compared to low light conditions (Quigg et al., 2012). These mechanisms may have occurred in *C. velia1* and *C. velia2* as a thermal acclimation response in our experiments, which is supported by the stable maximum pressure over PSII (Qm) across increasing temperature conditions. As well as the unknown chromerid, had lower photosynthetic efficiencies at the control 27°C compared to the two *C. velia* strains and *C. goreaui* and exhibited photophysiologies most similar to each other. Unlike the two *C. velia* isolates, *V. brassicaformis* was negatively affected by temperature, although low quantum yield values and pressure over PSII values stabilized after 22 days. *Vitrella brassicaformis* and *C. velia*, although closely related, differ in several features that could underpin their differences in photochemistry and response to elevated temperature. For example, *Chromera* spp. produce isofucoxanthin while *Vitrella* spp. rely on vaucheriaxathin (Moore et al., 2008; Oborník et al., 2012). While the roles of *V. brassicaformis* pigments are unknown, such differences could result in variations in light harvesting and non-photochemical quenching capacity. Morphologically, *V. brassicaformis* possesses a multiple-laminated cell wall and a large conspicuous pyrenoid that are absent in *C. velia* (Oborník et al., 2012). Although the cell wall layers are transparent, it is possible that the amount of light reaching the chloroplast is reduced. A large pyrenoid is present in cells of *V. brassicaformis* but not in *C. velia*. Pyrenoids are associated with the operation of a carbon concentrating mechanism and perhaps their presence in *V. brassicaformis* could account for differences in photosynthetic efficiencies through differences in carbon fixation. Finally, since the photosynthetic complexes are found within the thylakoid membrane in the chloroplast, changes in its composition under increased temperature may play a role in the thermal stability and/or acclimation of the light harvesting complexes (Tchernov et al., 2004). Specifically, galactolipids play an important role in thylakoid membrane stability (Wada and Murata, 2009) and mass spectrometry and transcriptomic analyses have shown that *C. velia* and *V. brassicaformis* differ in their galactolipid content (Khadka et al., 2014). This may result in differences in the stability of the photosynthetic membranes that could account for some of the observed differences in thermal tolerances between microalgal species.

### The Effect of Diuron and Diuron With Elevated Temperature on Free-Living Microalgal Photochemistry

*Cladocopium goreaui* was the most sensitive microalgal strain to diuron requiring 7.4 μg/L at the control temperature of 27°C for the inhibition of the effective quantum yield by 50% (EC50) while at 32°C the photosynthetic efficiency was already more than 50% inhibited before any diuron additions. Previous reported EC50 values testing the potency of diuron on free-living members of the Symbiodiniaceae have varied from 1.1 μg/L (van Dam et al., 2015) to 5.5 μg/L (Jones et al., 2003). The former experiment showed a small increase in diuron sensitivity for two Symbiodiniaceae species with elevated temperature. Other tropical microalgae at ambient 24–26°C have displayed EC50 values of between 2.1 and 4.4 μg/L (Magnusson et al., 2010; Mercurio et al., 2018). Although there is apparent variation in diuron sensitivity between tropical microalgae as well as between members of the Symbiodiniaceae, the concentration of diuron required to inhibit effective quantum yields by 50% are generally below 10 μg/L (but see Kottuparambil et al., 2013 where a cyanobacterium exhibited an EC50 of 7.15–14.8 μg/L) and similar in magnitude. The chromerids studied here are far more tolerant of diuron than *C. goreaui* and to our knowledge any other reported marine algae. The two strains of *C. velia, C. velia1*, and *C. velia2*, had EC50 values of 30 and 22 μg/L, respectively, and while they became more diuron-sensitive with elevated temperature, they still had high EC50 values of 17 and 18 μg/L, respectively. Despite their comparatively low sensitivity to diuron compared to *C. goreaui*, they were not able to match the unprecedented diuron tolerances of *V. brassicaformis* and the unknown chromerid that required 140-fold more diuron before the effective QYs were inhibited by 50%, with EC50 values of between 971 and 1,702 μg/L. Furthermore, while the effective QY of *V. brassicaformis* was inhibited by over 25% at 32°C, before the addition of diuron, the extreme diuron tolerance of the unknown chromerid was enhanced by elevated temperature.

The mechanism behind diuron toxicity on photosynthesis is well-understood. Diuron targets PSII in the chloroplasts of plants and algae by competing with the plastoquinone binding site of the D1 electron acceptor in PSII. This blocks the transfer of electrons, resulting in decreased photochemical efficiency (Van Rensen, 1989). Although the mechanisms underlying the differences in diuron tolerances between algae are unknown, we suggest several possibilities. Firstly, high concentrations of diuron may result in chronic photoinhibition (Jones, 2005) and algae differ in their abilities to combat photodamage through the rate of repair of their PSII reaction centers (Takahashi et al., 2004, 2009), as well as exhibiting differing abilities to combat the excessive production of reactive oxygen species that likely occur from a disruption in electron flow (Lesser, 1997; Suggett et al., 2008; McGinty et al., 2012; Wietheger et al., 2018). Secondly, the low photosynthetic efficiencies exhibited by *V. brassicaformis* and the unknown chromerid could result in a lower base level of reactive oxygen species production that could allow higher diuron (and temperature) tolerances here. Thirdly, differences in the binding site of the D1 protein could exist between the chromerids and other microalgae, as well as between species of chromerids, reducing or enhancing the effectiveness of diuron. It is possible that a mutation(s) on the psbA gene encoding the D1 protein could be a feature that explains both the diuron tolerance and lower photosynthetic efficiencies of *V. brassicaformis* and the unknown chromerid (without diuron) where photosynthesis is limited by the efficiency of electron binding and transfer in PSII. Finally, a plausible explanation as to the extraordinary diuron-tolerance of *V. brassicaformis* may simply result from their unique morphology. *V. brassicaformis* has up to a dozen cell walls layered upon each other (Oborník et al., 2012), which could act as a barrier against diuron. Indeed, Obernik and colleagues were unable to use DNA-staining dyes in their study on *V. brassicaformis*, due to the thickness and impermeability of the cell wall. This multi-layered cell wall could result in a trade-off between a reduced amount of light available for photosynthesis with protection against toxic chemicals, such as herbicides.

Uniquely, we observed that the unknown chromerid was less sensitive to diuron with elevated temperature, with a 61% increase in effective QY after 22 days of exposure to 32°C. It is possible that the binding affinity of diuron is reduced at elevated temperature with this strain through the D1 protein rate of turnover and/or repair or due to conformational changes to the D1 protein binding site (Jones and Kerswell, 2003). The culture history of this strain was different to the other chromerids, having spent 2.5 years growing under 30 μg/L of diuron. It is possible that over multiple generations, the unknown chromerid went through adaptive changes, that resulted in a lowered sensitivity to diuron and in parallel to elevated temperature; genetic correlations among traits can exist, whereby selection on one trait can elicit a response in another (Stanton et al., 2000; Etterson and Shaw, 2001; Blows and Hoffmann, 2005). Indeed, the photosynthetic apparatus that is the target of diuron is also sensitive to temperature and thus selection for herbicide tolerance may have correlated with increased thermal tolerance for the unknown chromerid.

### The Effect of Temperature and Diuron on Coral Larval Uptake of Microalgae

At least one cell of each microalgal species was observed within *Acropora tenuis* and *Acropora millepora* larval tissue during this experiment, which shows that the chromerids can enter and be hosted by the larvae. However, there was no apparent correlation between the temperature and diuron tolerance of the microalgal strains *in vitro* with their uptake by coral larvae or larval mortality in either coral species tested. At 27°C, the greatest microalgal uptake was *Cladocopium goreaui* by both coral species.

*Acropora tenuis* was more successful at taking up the chromerids compared to *A. millepora*, which displayed minimal colonization across all three temperatures and under diuron conditions. At 27°C in the absence of diuron, *C. goreaui* by far had the greatest colonization-ability with over 600 cells *per* larva. *C. velia1* and *C. velia2* were the next most taken-up by *A. tenuis* larvae, containing a mean of approximately six chromerid cells. These results are similar to those found by Cumbo and colleagues, where short-term inoculation (up to three days) for *A. digitifera* and *A. tenuis* larvae resulted in fewer than 10 *C. velia* cells *per* larva, compared to over 100 cells of *C. goreaui* per larva (Cumbo et al., 2013). In our study, the uptake of *V. brassicaformis* and the unknown chromerid was minimal. The low photosynthetic efficiencies observed for *V. brassicaformis* and the unknown chromerid *in vitro* could be a factor for their unfavorable symbiosis with coral larvae, where algae are unable to meet the energy demands of the larvae. Conversely, the higher uptake by *A. tenuis* larvae of *C. velia1* and *C. velia2* could be, in part, due to their higher photosynthetic efficiencies (which are comparable to those for *C. goreaui*).

There has been debate as to where the chromerids may lay on the scale from parasitism to mutalisism. Cumbo et al. (2013) indicated the potential for the chromerids to form a mutualistic relationship. These authors showed that three chromerid strains, including *C. velia1* and *C. velia2*, were located within the larval endoderm and ectoderm of corals, thus supporting a potentially mutualistic relationship. In contrast, Mohamed et al. (2018) investigated the transcriptomic response of *A. digitifera* larvae exposed to a different *C. velia* strain (Moore et al., 2008) up to 48 h after their introduction and concluded that *Chromera* is not a mutualist due to the transcriptomic resemblance of the coral larvae to a typical host-response to parasites or pathogens. These studies, along with ours, remain inconclusive as to the symbiotic relationship that the chromerids may have with corals. In our study, larval mortality was not significantly increased in the presence of chromerids, compared to *C. goreaui*. Furthermore, we did not observe increased uptake by the chromerids under elevated stress conditions (elevated temperature and/or the presence of diuron) that might have been indicative of a parasitic relationship.

Although larval uptake of microalgae was generally lowered in the presence of diuron. There was one major exception. *Acropora tenuis* hosted a significantly greater number of *C. goreaui* cells at 30°C in the presence of diuron, compared to no diuron. This result was unexpected as *C. goreaui* was the most sensitive algal strain to diuron *in vitro*, and these observations were not mirrored in *A. millepora* nor any previous studies on coral-algal symbioses under diuron conditions. The literature supports a negative effect of diuron on the coral-Symbiodiniaceae symbiosis. For example, significant reductions in effective QY were recorded at very low diuron concentrations (≥ 1 μg/L) across four GBR coral species, while higher diuron concentrations (10 μg/L and above) resulted in the significant loss of symbionts, and tissue retraction, causing coral bleaching (Jones et al., 2003; Cantin et al., 2007). The expulsion of symbionts from GBR coral *Pocillopora damicornis* was observed at 10 μg/L of diuron in both recruits and adult colonies (Negri et al., 2005). Generally, a detrimental and mostly additive effect of elevated temperature combined with diuron on corals and other marine organisms has been documented (Negri et al., 2011; van Dam et al., 2012, 2015; Wilkinson et al., 2017). Jones and Kerswell (2003) found that diuron phytotoxicity of Symbiodiniceae within the coral *Seriatopora hystrix* was less at 30°C than 20°C and the toxicity of diuron appears to increase on either side of the thermal optimum for marine species (Negri et al., 2011 and Wilkinson et al., 2017).

### Potential for the Chromerids as Alternative Symbionts Under Stressful Conditions

We found a highly variable response of free-living Symbiodiniaceae and chromerids to elevated temperature and diuron. The two *C. velia* strains showed comparatively high and sustained thermal tolerance and were moderately diuron-tolerant, while the *V. brassicaformis* strain exhibited high levels of diuron tolerance but was thermally sensitive. The unknown chromerid exhibited both thermal tolerance and an extreme diuron tolerance, with diuron being less toxic at elevated temperature. The photosynthetic advantages that the chromerids exhibited under stress *in vitro* did not affect the onset of symbiosis; larval uptake of algal cells was low regardless of temperature or diuron treatment. *Cladocopium goreaui* remained the microalgal strain most able to colonize both coral larval species. Thus, our results do not support the chromerids being dominant symbionts of the coral species tested. Given the low densities of the chromerids in the coral larvae, it is unlikely they contribute significantly to the nutrition of their coral host and our results suggest there is no value in the use of the chromerids as alternative/additional symbionts in an assisted evolution approach. However, it is possible that it takes longer than 14 days for corals to establish symbiosis with chromerids compared to Symbiodiniaceae. The previous two experiments investigating coral larval uptake of the chromerids only lasted 3 days (Cumbo et al., 2013) and 24 h (Mohamed et al., 2018), the latter of which did not quantitively measure algal uptake. Thus, it is unknown whether a longer duration of observation would result in greater *in hospite* densities. It is also possible that, like the Symbiodiniaceae, there is host-specificity in the symbioses with the chromerids. Indeed Cumbo et al. (2013) showed that *A. tenuis* larvae exposed to three strains of *C. velia* (including Mdig2 and Mdig3 used here) contained approximately six Mdig3 cells *per* larva, compared *C. goreaui* 160 cells *per* larva after three days post-introduction; results that are in line with *A. tenuis* uptake in this study, after 14 days. In contrast a different coral species, *A. digitifera*, hosted Mdig3 in numbers that exceeded the *in hospite* algal cell densities of *C. goreaui* after both 24 h and 2 days (Cumbo et al., 2013).

Finally, it is possible that a symbiont that may benefit one life stage of a coral, may not benefit another as the nutritional needs of corals are different throughout their life-history (Abrego et al., 2008; Jones et al., 2008; Quigley et al., 2016). All strains of chromerids so far have been isolated from adult coral colonies and it is possible that the uptake of the chromerids is unfavorable during the early life stages of corals. Indeed unlike adult corals, no apicomplexan DNA was detected among planulae from broadcast spawning coral species, however planulae from brooding coral species tested positive for apicomplexan DNA (Kirk et al., 2013a). We recommend future experiments should investigate uptake, symbiosis establishment and consequences of the chromerids across coral life stages and different species.

The differences in morphology, ultrastructure and genetics that have already been reported between *C. velia* and *V. brassicaformis* in the literature is large despite being represented by only a very small handful of isolates, with only one isolate of *V. brassicaformis* having been described (Oborník et al., 2012). Extensive phenotypic and physiological variation also exists within the Symbiodiniaceae, between genetically distinct species as well as within species (Parkinson et al., 2016; Suggett et al., 2017; Swain et al., 2017). This intra-species variation, along with the range of photophysiological responses to stressors that we observed in this study, indicates that with further discovery of chromerid strains there is likely additional diversity to be characterized. It is possible that there are other species within the phylum Chromerida that may function better as coral symbionts than those studied here.

From an assisted evolution stand point, this study has identified highly temperature tolerant and/or herbicide resistant microalgae that are found associated with corals and that can enter the coral host, albeit minimally. The Symbiodiniaceae symbiosis is in jeopardy in an era of rapid ocean warming and increased local stressors and understanding of the genetic architecture underpinning high stress tolerances in the chromerids may inform genetic engineering approaches targeted at enhancing thermal tolerance of Symbiodiniaceae symbionts. The insertion and expression of a new gene into the marine microalga *Chlorella* has been successfully used to achieve the expression of heterologous proteins for various applications (Yang et al., 2016). Such genetic manipulations may be applied to coral symbionts with the aim of enhancing their thermal tolerance.

Further studies should be carried out to isolate and characterize additional strains of the chromerids, and to investigate their relative ability to colonize all life stages of coral, their potential benefit to the coral host, and the cellular and genetic mechanisms that underpin any stress responses of these elusive algae.

## Author Contributions

LC, AN, and MO designed the experiment. LC carried out data collection and data analyses. LC wrote the first draft of the manuscript with input from AN and MO.

### Conflict of Interest Statement

The authors declare that the research was conducted in the absence of any commercial or financial relationships that could be construed as a potential conflict of interest.

## Supplementary Material

Figure S1 Neighbor-joining distance tree based on pairwise distances between the four chromerid microalgae. All bootstrap values (500 replicates) were < 36.

Figure S2 Microscopy images using differential interference contrast of free-living cultures of *Vitrella brassicaformis* **(A)**, the unknown chromerid **(B)**, *C. velia1* **(C)** and *C. velia2* **(D)**.

Table S1 Results from a BLASTn search of highly similar sequences using (database: nucleotide collection nr/nt). ^*^These isolates are described as *Chromera velia* in Genbank and in their respective publications, however they were later described *as Vitrella brassicaformis* by Oborník et al. (2012) and originate from the same strain, first isolated by Moore et al. (2008), and also used in this study.

Table S2 *In vitro* mean temperature, standard error, and degrees of freedom (df) that microalgal cultures experienced during the experiment for either 12 or 22 days.

Table S3 *In hospite* mean temperature, standard error, and degrees of freedom (df) that *Acropora tenuis* and *Acropora millepora* larvae experienced during the two-week infection experiment.

Table S4 Analysis of variance (ANOVA) output of linear mixed effects models for testing whether microalgal strain or temperature have a significant effect on photosynthetic traits; effective quantum yield (ΔF/F${}_{\text{m}}^{\prime }$), maximum quantum yield (F_v_/F_m_), maximum pressure over photosystem II (Q_m_) after 12 and 22 days of exposure to the different temperature conditions.

Table S5 Analysis of variance (ANOVA) output of general linear models testing whether microalgal strain, diuron, or their interaction has a significant effect on *Acropora tenuis* larval uptake, or larval mortality after 14 days of exposure to the different microalgal strains, diuron, and temperature conditions.

Table S6 Analysis of variance (ANOVA) output of general linear models testing whether microalgal strain, diuron or their interaction has a significant effect on *Acropora millepora* larval uptake, or larval mortality after 14 days of exposure to the different microalgal strains, diuron, and temperature conditions.

Table S7 The maximum and effective quantum yield values and pressure over photosystem II (Qm) of five microalgal strains after 12 and 22 days of exposure to different temperatures.

Table S8 The % inhibition of effective quantum yield values Dose-response of the percentage inhibition of the effective (Δ*F*/*Fm*′) quantum yields of five microalgal strains exposed for 48 h to diuron at 27°C. Inhibition is relative to the quantum yield values at 0 μg L-1of diuronand pressure over photosystem II (Qm) of five microalgal strains after 12 and 22 days of exposure to different temperatures.

Table S9 The % inhibition of effective quantum yield values Dose-response of the percentage inhibition of the effective (Δ*F*/*Fm*′) quantum yields of five microalgal strains exposed for 48 h to diuron at 27°C. Inhibition is relative to the quantum yield values at 0 μg L-1of diuronand pressure over photosystem II (Qm) of five microalgal strains after 12 and 22 days of exposure to different temperatures.

Table S10 The uptake of five algal strains by *Acropora tenuis* larvae at three different temperatures and with 0 or 30 μg/L of diuron.

Table S11 The uptake of five algal strains by *Acropora millepora* larvae at three different temperatures and with 0 or 30 μg/L of diuron.
