# Supplementary figures and images for: Thermal and Herbicide Tolerances of Chromerid Algae and Their Ability to Form a Symbiosis With Corals
Source: Front Microbiol. 2019 Feb 12;10:173. doi: 10.3389/fmicb.2019.00173 (PMC6379472; doi:10.3389/fmicb.2019.00173)

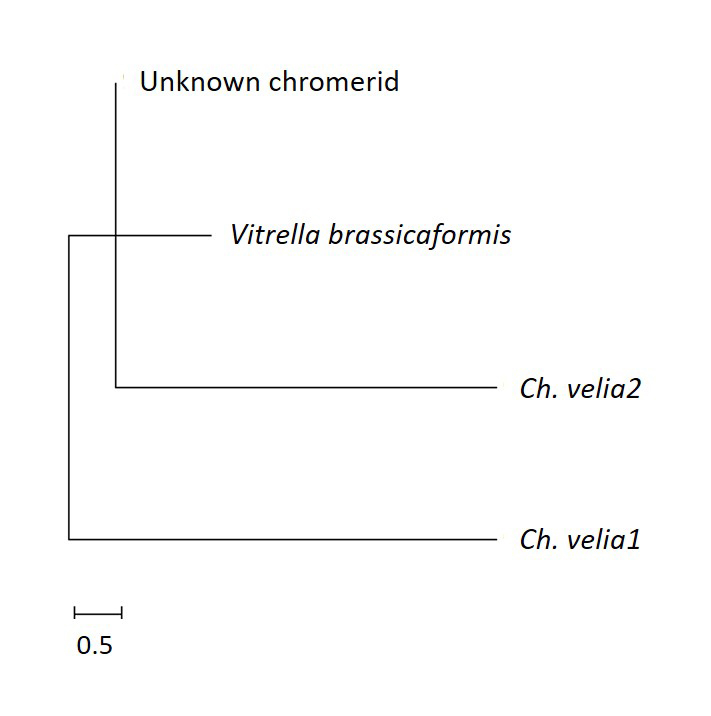

Supplement: Figure S1 — Neighbor-joining distance tree based on pairwise distances between the four chromerid microalgae. All bootstrap values (500 replicates) were < 36. [file Image_1.JPEG]

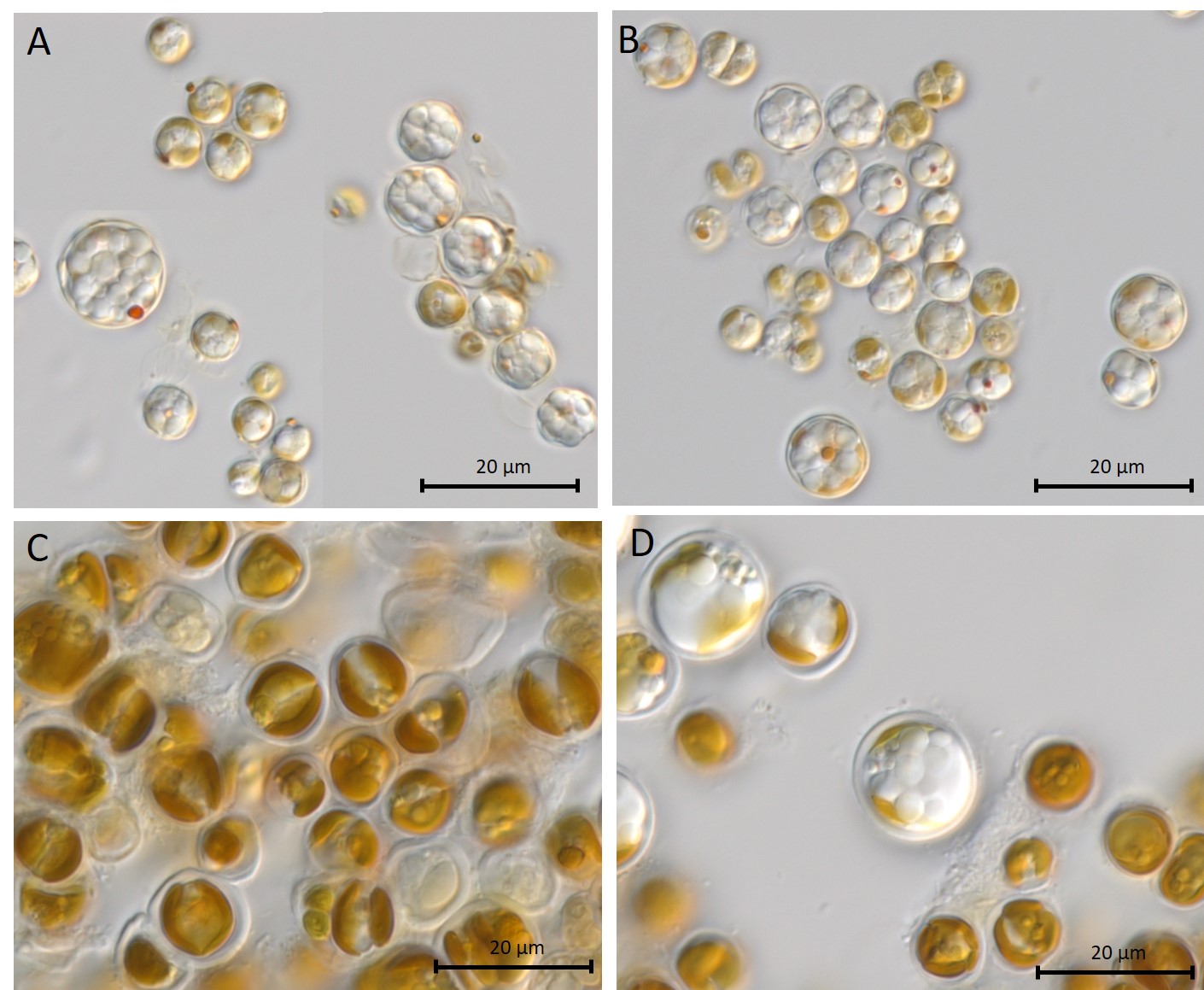

Supplement: Figure S2 — Microscopy images using differential interference contrast of free-living cultures of Vitrella brassicaformis (A), the unknown chromerid (B), C. velia1 (C) and C. velia2 (D). [file Image_2.JPEG]
