# Supplementary material for: Thermal and Herbicide Tolerances of Chromerid Algae and Their Ability to Form a Symbiosis With Corals
Source: Front Microbiol. 2019 Feb 12;10:173. doi: 10.3389/fmicb.2019.00173 (PMC6379472; doi:10.3389/fmicb.2019.00173)
Supplement: Table S1 — Results from a BLASTn search of highly similar sequences using (database: nucleotide collection nr/nt). *These isolates are described as Chromera velia in Genbank and in their respective publications, however they were later described as Vitrella brassicaformis by Oborník et al. (2012) and originate from the same strain, first isolated by Moore et al. (2008), and also used in this study. [file Table_1.DOCX]

**Table S1.** Results from a BLASTn search of highly similar sequences using (database: nucleotide collection nr/nt). *these isolates are described as *Chromera velia* in Genbank and in their respective publications, however they were later described *as Vitrella brassicaformis* by Obornik et al. (2012) and originate from the same strain, first isolated by Moore et al. (2008) and also used in this study.

| Accession number | Species name | Isolate | Clone | Score | Query cover | Expect value | Publication |
| --- | --- | --- | --- | --- | --- | --- | --- |
| JN986791.1 | *Chromera velia* | Mdig4 |  | 3070 | 100% | 0 | unpublished (2012) |
| DQ174731.1 | **Vitrella brassicaformis* | CMS22 |  | 3066 | 100% | 0 | Moore et al. 2008 |
| JN986790.1 | *Chromera velia* | Mdig3 |  | 6053 | 100% | 0 | Cumbo et al. 2013 |
| JN986789.1 | *Chromera velia* | Mdig2 |  | 6048 | 100% | 0 | Cumbo et al. 2013 |
| JN986792.1 | *Chromera velia* | Mdig5 |  | 3042 | 100% | 0 | unpublished (2012) |
| JN935832.1 | **Vitrella brassicaformis* | CvLp_vc08/1 | JS494 | 3009 | 98% | 0 | Morin-Adeline et al. 2012 |
| JN935830.1 | **Vitrella brassicaformis* | CvLp_vc08/1 | JS492 | 3009 | 98% | 0 | Morin-Adeline et al. 2012 |
| JN935829.1 | *Vitrella brassicaformis* | CvLp_vc08/1 | JS491 | 3009 | 98% | 0 | Morin-Adeline et al. 2012 |
| JN986788.1 | *Chromera velia* | Mdig1 |  | 3005 | 100% | 0 | Cumbo et al. 2013 |
| JN935834.1 | **Vitrella brassicaformis* | CvLp_vc08/1 | JS496 | 2882 | 98% | 0 | Morin-Adeline et al. 2012 |
