# Supplementary material for: Thermal and Herbicide Tolerances of Chromerid Algae and Their Ability to Form a Symbiosis With Corals
Source: Front Microbiol. 2019 Feb 12;10:173. doi: 10.3389/fmicb.2019.00173 (PMC6379472; doi:10.3389/fmicb.2019.00173)
Supplement: Table S2 — In vitro mean temperature, standard error, and degrees of freedom (df) that microalgal cultures experienced during the experiment for either 12 or 22 days. [file Table_2.DOCX]

**Table S2**. *In vitro* mean temperature, standard error and degrees of freedom (df) that microalgal cultures experienced during the experiment for either 12 or 22 days.

| Temperature exposure (days) | Temperature (°C) | Mean | Standard error (±) | df |
| --- | --- | --- | --- | --- |
| 12 | 27 | 26.82 | 0.004 | 1728 |
| 12 | 30 | 30.01 | 0.003 | 1728 |
| 12 | 31 | 31.12 | 0.012 | 1728 |
| 12 | 32 | 31.91 | 0.011 | 1728 |
| 22 | 27 | 26.76 | 0.004 | 3168 |
| 22 | 30 | 30.01 | 0.002 | 3168 |
| 22 | 31 | 31.10 | 0.007 | 3168 |
| 22 | 32 | 31.91 | 0.006 | 3168 |
