# Supplementary material for: Thermal and Herbicide Tolerances of Chromerid Algae and Their Ability to Form a Symbiosis With Corals
Source: Front Microbiol. 2019 Feb 12;10:173. doi: 10.3389/fmicb.2019.00173 (PMC6379472; doi:10.3389/fmicb.2019.00173)
Supplement: Table S3 — In hospite mean temperature, standard error, and degrees of freedom (df) that Acropora tenuis and Acropora millepora larvae experienced during the two-week infection experiment. [file Table_3.DOCX]

**Table S3**. *In hospite* mean temperature, standard error and degrees of freedom (df) that *Acropora tenuis* and *Acropora millepora* larvae experienced during the two-week infection experiment.

| Species | Temperature (°C) | Mean | Standard error (±) | df |
| --- | --- | --- | --- | --- |
| *A. tenuis* | 27 | 26.66 | 0.008 | 2016 |
| *A. tenuis* | 30 | 30.06 | 0.002 | 2016 |
| *A. tenuis* | 31 | 31.12 | 0.010 | 2016 |
| *A. millepora* | 27 | 26.83 | 0.005 | 2016 |
| *A. millepora* | 30 | 30.06 | 0.002 | 2016 |
| *A. millepora* | 31 | 31.12 | 0.002 | 2016 |
