# Supplementary material for: Thermal and Herbicide Tolerances of Chromerid Algae and Their Ability to Form a Symbiosis With Corals
Source: Front Microbiol. 2019 Feb 12;10:173. doi: 10.3389/fmicb.2019.00173 (PMC6379472; doi:10.3389/fmicb.2019.00173)
Supplement: Table S4 — Analysis of variance (ANOVA) output of linear mixed effects models for testing whether microalgal strain or temperature have a significant effect on photosynthetic traits; effective quantum yield (ΔF/Fm′), maximum quantum yield (Fv/Fm), maximum pressure over photosystem II (Qm) after 12 and 22 days of exposure to the different temperature conditions. [file Table_4.DOCX]

**Table S4.** Analysis of variance (ANOVA) output of linear mixed effects models for testing whether microalgal strain or temperature have a significant effect on photosynthetic traits; effective quantum yield (ΔF/F_m_’), maximum quantum yield (F_v_/F_m_), maximum pressure over photosystem II (Q_m_) after 12 and 22 days of exposure to the different temperature conditions.

| Trait | Days of exposure | Source | DF | F | p-value |
| --- | --- | --- | --- | --- | --- |
| ΔF/F_m_’ | 12 | Strain | 40 | 352.8 | <0.0001 |
|  | 12 | Temperature | 40 | 28.4 | <0.0001 |
|  | 12 | Strain:temperature | 40 | 39.7 | <0.0001 |
| F_v_/F_m_ | 12 | Strain | 40 | 294.5 | <0.0001 |
|  | 12 | Temperature | 40 | 18.5 | <0.0001 |
|  | 12 | Strain:temperature | 40 | 5.1 | <0.0001 |
| Q_m_ | 12 | Strain | 40 | 31.7 | <0.0001 |
|  | 12 | Temperature | 40 | 4.6 | 0.0076 |
|  | 12 | Strain:temperature | 40 | 10.4 | <0.0001 |
| ΔF/F_m_’ | 22 | Strain | 40 | 791.0 | <0.0001 |
|  | 22 | Temperature | 40 | 251.8 | <0.0001 |
|  | 22 | Strain:temperature | 40 | 110.4 | <0.0001 |
| F_v_/F_m_ | 22 | Strain | 40 | 806.1 | <0.0001 |
|  | 22 | Temperature | 40 | 520.7 | <0.0001 |
|  | 22 | Strain:temperature | 40 | 171.3 | <0.0001 |
| Q_m_ | 22 | Strain | 40 | 36.6 | <0.0001 |
|  | 22 | Temperature | 40 | 3.6 | 0.0211 |
|  | 22 | Strain:temperature | 40 | 4.6 | <0.0001 |
