# Supplementary material for: Thermal and Herbicide Tolerances of Chromerid Algae and Their Ability to Form a Symbiosis With Corals
Source: Front Microbiol. 2019 Feb 12;10:173. doi: 10.3389/fmicb.2019.00173 (PMC6379472; doi:10.3389/fmicb.2019.00173)
Supplement: Table S5 — Analysis of variance (ANOVA) output of general linear models testing whether microalgal strain, diuron, or their interaction has a significant effect on Acropora tenuis larval uptake, or larval mortality after 14 days of exposure to the different microalgal strains, diuron, and temperature conditions. [file Table_5.DOCX]

**Table S5.** Analysis of variance (ANOVA) output of general linear models testing whether microalgal strain, diuron or their interaction has a significant effect on *Acropora tenuis* larval uptake or larval mortality after 14 days of exposure to the different microalgal strains, diuron and temperature conditions.

| Train | Temperature (°C) | Source | DF | Chisq | p-value | |
| --- | --- | --- | --- | --- | --- | --- |
| Larval uptake | 27 | Strain | 4 | 49819 | <0.0001 | |
|  |  | Diuron | 1 | 8074 | <0.0001 | |
|  |  | Strain:diuron | 4 | 25 | <0.0001 | |
|  | 30 | Strain | 4 | 1352 | <0.0001 | |
|  |  | Diuron | 1 | 284 | <0.0001 | |
|  |  | Strain:diuron | 4 | 427 | <0.0001 | |
|  | 31 | Strain | 4 | 62 | <0.0001 | |
|  |  | Diuron | 1 | 2.1 | 0.145 | |
| Larval mortality | 27 | Strain | 4 | 41 | <0.0001 | |
|  |  | Diuron | 1 | 4.0 | 0.047 |  |
|  | 30 | Strain | 4 | 8.8 | 0.066 |  |
|  |  | Diuron | 1 | 4.2 | 0.040 |  |
|  | 31 | Strain | 4 | 44 | <0.0001 |  |
|  |  | Diuron | 1 | 6.1 | 0.013 |  |
|  |  | Strain:diuron | 4 | 25 | <0.0001 |  |
|  |  |  |  |  |  |  |
|  |  |  |  |  |  |  |
