# Supplementary material for: Thermal and Herbicide Tolerances of Chromerid Algae and Their Ability to Form a Symbiosis With Corals
Source: Front Microbiol. 2019 Feb 12;10:173. doi: 10.3389/fmicb.2019.00173 (PMC6379472; doi:10.3389/fmicb.2019.00173)
Supplement: Table S6 — Analysis of variance (ANOVA) output of general linear models testing whether microalgal strain, diuron or their interaction has a significant effect on Acropora millepora larval uptake, or larval mortality after 14 days of exposure to the different microalgal strains, diuron, and temperature conditions. [file Table_6.DOCX]

**Table S6.** Analysis of variance (ANOVA) output of general linear models testing whether microalgal strain, diuron or their interaction has a significant effect on *Acropora millepora* larval uptake or larval mortality after 14 days of exposure to the different microalgal strains, diuron and temperature conditions.

| Trait | Temperature (°C) | Source | DF | Chisq | p-value |
| --- | --- | --- | --- | --- | --- |
| Larval uptake | 27 | Strain | 4 | 32594 | <0.0001 |
|  |  | Diuron | 1 | 5148 | <0.0001 |
|  |  | Strain:diuron | 4 | 90 | <0.0001 |
|  | 30 | Strain | 4 | 435 | <0.0001 |
|  |  | Diuron | 1 | 3.6 | 0.056 |
|  |  | Strain:diuron | 4 | 27 | <0.0001 |
|  | 31 | Strain | 4 | 59 | <0.0001 |
|  |  | Diuron | 1 | 26 | <0.0001 |
|  |  | Strain:diuron | 4 | 10 | 0.039 |
| Larval mortality | 27 | Strain | 4 | 27 | <0.0001 |
|  |  | Diuron | 1 | 23 | <0.0001 |
|  | 30 | Strain | 4 | 5.2 | 0.265 |
|  |  | Diuron | 1 | 5.5 | 0.019 |
|  |  | Strain:diuron | 4 | 9.5 | 0.050 |
|  | 31 | Strain | 4 | 7.0 | 0.135 |
|  |  | Diuron | 1 | 0.74 | 0.390 |
|  |  | Strain:diuron | 4 | 9.8 | 0.044 |
